# Nationwide implementation of the international multidisciplinary best-practice for locally advanced pancreatic cancer (PREOPANC-4): study protocol

**DOI:** 10.1186/s12885-025-13554-w

**Published:** 2025-02-19

**Authors:** T. F. Stoop, L. W. F. Seelen, F. R. van ’t Land, A. C. van der Hout, J. C. M. Scheepens, M. Ali, A. M. Stiggelbout, B. M. van der Kolk, B. A. Bonsing, D. J. Lips, D. J. A. de Groot, E. van Veldhuisen, E. D. Kerver, E. R. Manusama, F. Daams, G. Kazemier, G. A. Cirkel, G. van Tienhoven, G. A. Patijn, H. N. Lelieveld-Rier, I. H. de Hingh, I. E. G. van Hellemond, J. H. Wijsman, J. I. Erdmann, J. S. D. Mieog, J. de Vos-Geelen, J. W. B. de Groot, K. R. D. Lutchman, L. J. Mekenkamp, L. W. Kranenburg, L. P. M. Beuk, M. W. Nijkamp, M. den Dulk, M. B. Polée, M. Y. V. Homs, M. L. Wumkes, M. W. J. Stommel, O. R. Busch, R. F. de Wilde, R. T. Theijse, S. A. C. Luelmo, S. Festen, T. L. Bollen, U. P. Neumann, V. E. de Meijer, W. A. Draaisma, B. Groot Koerkamp, I. Q. Molenaar, C. L. Wolfgang, M. Del Chiaro, M. G. H. Katz, T. Hackert, J. A. C. Rietjens, J. W. Wilmink, H. C. van Santvoort, C. H. J. van Eijck, M. G. Besselink, for the Dutch Pancreatic Cancer Group, A. Bruynzeel, A. Vlijm, A. van Asseldonk, B. Zonderhuis, A.A. Javed, A. Sterk, A. Schoorlemmer, A. Stam, C.Y. Nio, E. van Alphen, H.W.M. van Laarhoven, H. van Veenendaal, I. Griffioen, I.F. Rompen, J.M. Klaase, M. Los, M.F.M.A. Marting, M.S.L. Liem, J. Peters, L.J. Peters, Linda Garms, M. Walma, L.H.J. Brada, M. Seijbel, N.D. Hildebrand, N. Michiels, R. Bax, R.M. van Dam, S. Achten, S. Bouwense, S. Augustinus, S. Rötgerink, T.W. van Ravens, V.B. Nieuwenhuijs, W.W. te Riele

**Affiliations:** 1 Amsterdam UMC, Location University of Amsterdam, Department of Surgery, Amsterdam, the Netherlands; 2 Cancer Center Amsterdam, Amsterdam, the Netherlands; 3 Division of Surgical Oncology, Department of Surgery, University of Colorado Anschutz Medical Campus, Aurora, CO USA; 4 Department of Surgery, Regional Academic Cancer Center Utrecht, University Medical Center Utrecht / St. Antonius Hospital Nieuwegein, Nieuwegein & Utrecht, the Netherlands; 5 Department of Surgery and Pulmonology, Erasmus MC Cancer Institute, University Medical Center, Rotterdam, the Netherlands; 6 Department of Public Health, Erasmus MC, University Medical Center Rotterdam, Rotterdam, the Netherlands; 7 Amsterdam UMC, Location University of Amsterdam, Department of Radiation Oncology, Amsterdam, the Netherlands; 8 Department of Biomedical Data Sciences, Leiden University Medical Center, Leiden, the Netherlands; 9 Department of Medical Oncology, Amphia Hospital, Breda, the Netherlands; 10 Amsterdam UMC, Location University of Amsterdam, Department of Medical Oncology, Amsterdam, the Netherlands; 11 Department of Surgery, Leiden University Medical Center, Leiden, the Netherlands; 12 Department of Surgery, Radboud University Medical Center, Nijmegen, the Netherlands; 13 Department of Medical Oncology, Isala Oncology Center, Isala, Zwolle, the Netherlands; 14 Department of Surgery, Medisch Spectrum Twente, Enschede, the Netherlands; 15 Department of Medical Oncology, University Medical Center Groningen, University of Groningen, Groningen, the Netherlands; 16 Department of Medical Oncology, OLVG, Amsterdam, the Netherlands; 17 Department of Surgery, Medical Center Leeuwarden, Leeuwarden, the Netherlands; 18 Amsterdam UMC, Location Vrije Universiteit, Department of Surgery, Amsterdam, the Netherlands; 19 Department of Medical Oncology, Regional Academic Cancer Center Utrecht, St. Antonius Hospital Nieuwegein, University Medical Center Utrecht, Nieuwegein & Utrecht, the Netherlands; 20 Department of Surgery, Isala, Zwolle, the Netherlands; 21 Department of Medical Oncology, Catharina Hospital, Eindhoven, the Netherlands; 22 Department of Surgery, Amphia Hospital, Breda, the Netherlands; 23 Department of Internal Medicine, Division of Medical Oncology, GROW – Research Institute for Oncology & Reproduction, Maastricht University Medical Center, Maastricht, the Netherlands; 24 Department of Medical Oncology, Medisch Spectrum Twente, Enschede, the Netherlands; 25 Department of Psychiatry, Erasmus University Medical Center, Rotterdam, the Netherlands; 26 Department of Surgery, University of Groningen and University Medical Center Groningen, Groningen, the Netherlands; 27 Department of Surgery, Maastricht University Medical Center, Maastricht, the Netherlands; 28 Nutrim School for Nutrition and Translational Research in Metabolism, Maastricht, the Netherlands; 29 Department of Medical Oncology, Medical Center Leeuwarden, Leeuwarden, the Netherlands; 30 Department of Medical Oncology, Erasmus MC Cancer Institute, Rotterdam, the Netherlands; 31 Department of Medical Oncology, Jeroen Bosch Hospital, ‘s , Hertogenbosch, the Netherlands; 32 Department of Medical Oncology, Leiden University Medical Center, Leiden, the Netherlands; 33 Department of Surgery, OLVG, Amsterdam, the Netherlands; 34 Department of Radiology, St. Antonius Hospital, Nieuwegein, the Netherlands; 35 Department of Surgery, Jeroen Bosch Hospital, ‘s, Hertogenbosch, the Netherlands; 36 Department of Surgery, NYU Langone Health, New York, NY USA; 37 Department of Surgical Oncology, The University of Texas MD Anderson Cancer Center, Houston, TX USA; 38 Department of General, Visceral and Thoracic Surgery, University Hospital Hamburg-Eppendorf, Hamburg, Germany; 39 Department of General, Visceral and Transplantation Surgery, Heidelberg University Hospital, Heidelberg, Germany; 40 Department of Design, Organisation and Strategy, Faculty of Industrial Design Engineering, Delft University of Technology, Delft, the Netherlands; 41 Department of Surgery, University Hospital Essen, Essen, Germany; 42 Amsterdam UMC, Location University of Amsterdam, Department of Surgery, De Boelelaan 1117 (ZH-7F), Amsterdam, HV 1081 the Netherlands; 43 Department of Surgery, Catharina Hospital, Eindhoven, the Netherlands

**Keywords:** Locally advanced pancreatic cancer, Induction therapy, Surgery, Implementation program, The Netherlands

## Abstract

**Background:**

The introduction of (m)FOLFIRINOX and gemcitabine-nab-paclitaxel has changed the perspective for patients with locally advanced pancreatic cancer (LAPC). Consequently, in experienced centres 23% of patients with LAPC undergo a resection with 5-year overall survival (OS) rates of up to 25%. In the Netherlands, the nationwide resection rate for LAPC remains low at 8%. The PREOPANC-4 program aims for a nationwide implementation of the international multidisciplinary best-practice to improve patient outcome.

**Methods:**

Nationwide program implementing the international multidisciplinary best-practice for LAPC. In the training phase, multidisciplinary and surgical webinars are given by 4 international experts, leading to a clinical protocol, followed by surgical off-site and on-site proctoring sessions. In the implementation phase, the clinical protocol will be implemented in all centres, including a nationwide expert panel (2022–2024). Healthcare professionals will be trained in shared decision-making. Consecutive patients diagnosed with pathology-proven LAPC (i.e., arterial involvement > 90° and/or portomesenteric venous > 270° involvement or occlusion [DPCG criteria]) are eligible. Primary outcomes are median and 5-year OS from diagnosis, resection rate, in-hospital/30-day mortality and major morbidity (i.e., Clavien-Dindo grade ≥ IIIa), and radical resection (R0) rate. Secondary outcomes include quality of life, functioning, side effects, and patients’ healthcare satisfaction in all included patients. Outcomes will be compared with patients with borderline resectable pancreatic cancer (BRPC) treated with neoadjuvant FOLFIRINOX in the PREOPANC-2 trial (EudraCT: 2017–002036-17) and a historical cohort of patients with LAPC from the PACAP registry (NCT03513705). The existing prospective LAPC Registry and PACAP PROMs (NCT03513705) will be used for data collection. In qualitative interviews, treatment preferences, values, and experiences of LAPC patients, their relatives, and healthcare professionals will be assessed for the development of shared decision-making supportive tools. It is hypothesized that the program will double the nationwide LAPC resection rate to 16% with major morbidity < 50% and mortality ≤ 5%, and OS following resection similar to that observed in patients with BRPC.

**Discussion:**

The PREOPANC-4 program aims to safely implement the international multidisciplinary best-practice for LAPC leading to benchmark outcomes for both short-term morbidity, mortality, and OS.

**Trial registration:**

PREOPANC-4 program was registered at ClinicalTrials.gov (NCT05524090) on September 1, 2022.

**Supplementary Information:**

The online version contains supplementary material available at 10.1186/s12885-025-13554-w.

## Introduction

Pancreatic ductal adenocarcinoma (hereafter: *pancreatic cancer*) is predicted to become the second cause of cancer-related death this decade, with a current 2.1% life time risk of developing pancreatic cancer in Western Europe [1, 2]. Chemotherapy in combination with surgical resection is currently the cornerstone of treatment [3]. Unfortunately, about two-thirds of patients with non-metastatic pancreatic cancer have extensive vascular involvement (i.e., locally advanced pancreatic cancer [LAPC]) that prohibits an oncologically meaningful resection [4]. For decades, either palliative systemic therapy or best supportive care was the standard of care for these patients, associated with limited overall survival (OS) of only several months [5]. The introduction of (m)FOLFIRINOX (i.e., a [modified] combination of 5-fluorouracil, leucovorin, irinotecan, and oxaliplatin) and gemcitabine with nab-paclitaxel [6–8] has changed the perspective for patients with LAPC due to improved disease control [5]. Consequently, together with improvements in response evaluation [5, 9, 10], in international expert centres up to 23% of patients undergo surgical resection [11], associated with acceptable morbidity and mortality [12–14] and 5-year OS rates of up to 25% [14–16].

Although data on surgery in patients with LAPC is surprisingly consistent, some reluctance persists in the Netherlands [5]. A recent Dutch nationwide observational study demonstrated that the resection rate of patients with LAPC is only 8% despite acceptable morbidity, mortality, and OS [17, 18]. It is unlikely that this substantial difference in resection rates between the Netherlands and the international literature (8% *versus*23%) is caused solely by referral and publication bias [19], given that the Dutch guideline has more restrictive resectability criteria [20, 21] than international guidelines [5]. Instead, differences in induction therapy regimens, subsequent response evaluation, perioperative patient selection, and surgical techniques are a more likely explanation [17, 18]. There seems to be room for improvement in the treatment of patients with LAPC aiming to achieve improved OS. Therefore, the nationwide PREOPANC-4 program was initiated to improve and standardize multidisciplinary care for patients with LAPC [22, 23]. This program will implement the international best-practice for LAPC in the Netherlands in close collaboration with 4 international experts, aiming to improve the OS outcome safely and patient-centered.

## Methods

This study protocol is reported following the 2013 Standard Protocol Items: Recommendations for Interventional Trials (SPIRIT) guideline. See Appendix 1 for the SPIRIT checklist [24].

### Study design

The PREOPANC-4 training phase started in 2021 after which the 3-year implementation will take place from January 2022 until December 2024, followed by a follow-up of 5 years until December 2029.

### Participants

This prospective nationwide implementation program will be performed in the centres of the Dutch Pancreatic Cancer Group (DPCG) [25]. Four experts from international expert centres were invited as proctors to develop and participate in the PREOPANC-4 program: M. Del Chiaro (University of Colorado Anschutz Medical Campus, United States), T. Hackert (University of Heidelberg & University of Hamburg-Eppendorf, Germany), C.L. Wolfgang (NYU Langone Health, United States), and M.H.G. Katz (The University of Texas MD Anderson Cancer Center, United States).

### Design

#### Training phase

The training phase includes a theoretical and practical training program overseen by the 4 experts. Participants in this training program are the multidisciplinary teams from all 7 Dutch university medical centres. The three highest volume centres in pancreatic surgery including LAPC surgery (i.e., Amsterdam UMC, ErasmusMC, RACU) [18] will function as referral centres for LAPC surgery during the implementation. The 4 other university medical centres (i.e., UMCG, MUMC + , LUMC, Radboud UMC) will participate in the training program, since the DPCG is working towards a situation in the Netherlands in which LAPC surgery is mainly concentrated in 5 centres.

The multidisciplinary training program comprises the following 3 steps: (1) multidisciplinary training program; (2) surgical technique training and off-site proctoring; and (3) on-site proctoring.

- *Step 1—Multidisciplinary training program*: The proctors will educate the Dutch multidisciplinary teams (including medical and radiation oncologists, gastroenterologists, surgeons, radiologists, and pathologists specialized in pancreatic cancer) from those selected Dutch centres mentioned-above on induction therapy regimens and patient selection; response evaluation; indications for surgery, based on scientific evidence and expert opinion. The proctors will organize additional surgical webinars for pancreatic surgeons to discuss intraoperative decision-making and surgical techniques.
- *Step 2—Surgical technique training and off-site proctoring:* The proctors will organize a training session in the Skills Lab & Simulation Center from the ErasmusMC (Rotterdam) for pancreatic surgeons from the 7 university medical centres. This training session consists of lectures and practical skills training on the approach of peripancreatic vasculature; (contra-)indications for and techniques of arterial divestment; pitfalls and (contra-)indications for portomesenteric venous and arterial resections (and reconstructions); and perioperative monitoring and anti-coagulants regimens. Subsequently, pancreatic surgeons from each of the 3 high-volume Dutch centres will be surgically trained via off-site proctoring during several live LAPC surgeries in the proctors’ centres.
- *Step 3—On-site proctoring:* The pancreatic surgeons from the 3 highest-volume Dutch centres will be proctored in their institution by the proctor(s) during LAPC surgery. Pancreatic surgeons from the 4 observing university medical centres will join these sessions.

When expanding the indications for surgery, patient-centered care is of vital importance [26–28]. Therefore, healthcare providers (i.e., pancreatic surgeons, medical oncologists, and nurse specialists) from the 3 LAPC referral centres will be trained in shared decision-making. This training will follow the methods from an ongoing randomized controlled trial (Netherlands Trial Registry: NL9647) [29], including an e-learning training module, reflection on feedback reports from recorded consultations, and individual coaching [30].

#### Implementation phase

**Fig. 1:**
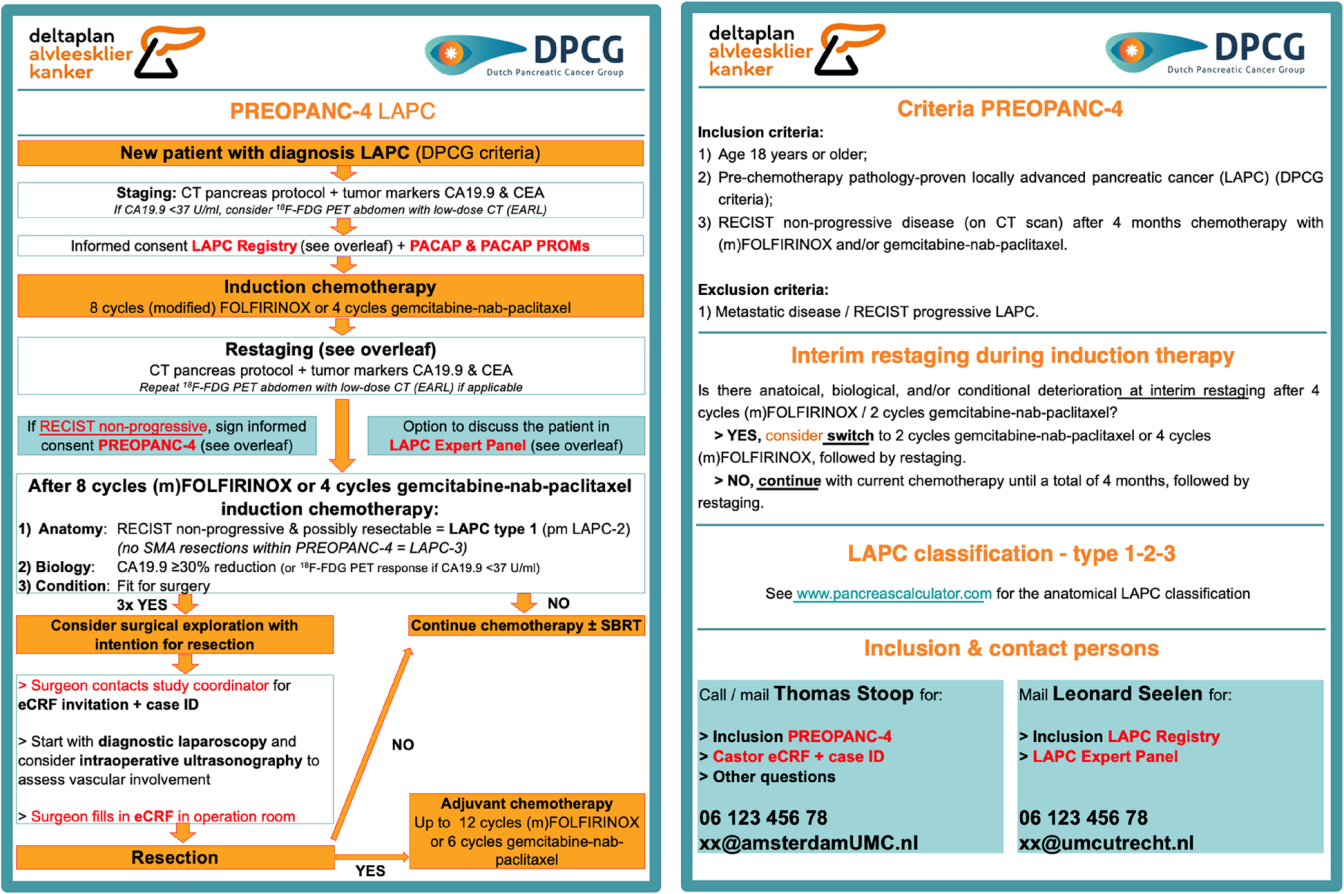
Clinical work-up. *LAPC,* locally advanced pancreatic cancer; *DPCG,* Dutch Pancreatic Cancer Group; *CT,* computed tomography; *CA19-9,* carbohydrate antigen 19–9; *CEA,* carcinoembryonic antigen; *U,* units; *ml,*millilitres [18]; *F-FDG PET/CT*, 2-deoxy-2-[fluorine-18]-fluoro-D-glucose positron emission tomography; *PACAP,* PAncreatic CAncer Project (NCT03513705); *PROMs,* patient-reported outcome measures; *RECIST,* Response Evaluation Criteria in Solid Tumours [31]; *eCRF,* electronic case report form; *SBRT,* stereotactic body radiotherapy. NB. The LAPC 1–2–3classification concerns the anatomy-based classification system from Johns Hopkins University [32]

Based on the multidisciplinary and surgical training program, a clinical work-up is designed in collaboration with the principal investigators and proctors. In this work-up, the induction therapy strategies, response evaluation, and subsequent selection for surgery will be standardized. Importantly, this work-up is flexible, but can be modified based on new evidence and/or clinical experiences. This clinical work-up representing the so-called ‘international best practice’ for patients with LAPC will be implemented within all 14 participating DPCG centres. See Fig. 1 for the clinical work-up. The implementation will be initiated via a kick-off meeting with the multidisciplinary team at each participating centre.

An online national LAPC expert panel will take place via virtual meetings bi-weekly throughout the 3-year implementation phase [17, 33]. Here, clinicians from the participating centres can present LAPC cases at time of (interim) restaging during or following induction chemotherapy to discuss treatment strategies, including the indications for surgery and surgical techniques. A second (international) expert panel will be scheduled with the international proctors to discuss specific cases. The national LAPC expert panel comprises surgeons from the 7 university medical centres, a medical oncologist, and an abdominal radiologist examining cross-sectional imaging from each patient.

An online monthly meeting will be organized with the international proctors to evaluate LAPC cases that have undergone surgery, aiming to exchange experiences and to learn from each other. Furthermore, an annual meeting with the international proctors will be organized to evaluate the implementation strategy and surgical outcome. Eventually, the clinical work-up can be optimized based on new evidence. In this case, the updated implementation strategy will be available on the DPCG website and spread among local study members and clinicians via the local principal investigators with the use of printed pocked cards containing the clinical work-up (see Fig. 1).

Together with the Dutch and international multidisciplinary teams, the study coordinator will develop a supportive guideline for the PREOPANC-4 program, comprising all information from the webinars and live operations. This supportive guideline will be used as reference during the PREOPANC-4 program. Based on the surgical webinars, several surgical ‘lead principles’ are established for the implementation phase. Arterial divestment in patients with arterial involvement is performed if there is no evidence of arterial wall ingrowth on preoperative cross-sectional imaging and based on intraoperatively assessment (i.e., no string sign, negative fresh frozen biopsies, peri-arterial tissue can be peeled off the artery without significant resistance) [34, 35]. Importantly, tumour in-growth in the superior mesenteric artery (SMA) on preoperative cross-sectional imaging (i.e., string sign) [35] requiring an SMA resection to achieve a radical resection is considered to be a contraindication for surgery in the PREOPANC-4 program, given the associated high short-term mortality of 7% in even very high-volume experienced centres [36–38]. Nevertheless, even an arterial divestment can still result in arterial damage (e.g., hemorrhage, dissection) necessitating an arterial resection, so surgeons should be prepared and trained for such events and procedures [39, 40]. Concerning portomesenteric venous involvement requiring a segmental portomesenteric venous resection (PVR), the need for an interposition graft is reduced by performing a Cattell-Braasch maneuver, allowing complete mobilization of the bowel and mesentery [41]. Other methods to enhance the length for a tension-free end-to-end portomesenteric reconstruction is mobilization of the liver, resecting the splenic vein, and dissecting the portal vein towards its bifurcation. If an interposition graft is still needed for a tension-free anastomosis, an autologous graft, tubularized Bovine patch, or cadaveric graft is preferred over alloplastic grafts that have an increased risk of portomesenteric venous thrombosis [42]. A mesocaval shunt or meso-portal bypass at the start of the procedure should be considered in case of extensive collaterals due to venous occlusion by a thrombus or tumour compression. In this way, the portal hypertension through the venous collaterals is released, minimizing the risk of major bleeding during the dissection phase. Once the specimen is out, the portomesenteric continuity can be restored by end-to-end reconstruction or shortening the graft that was used for the shunt or bypass [43–45].

### Objectives and hypotheses

It is hypothesized that the PREOPANC-4 program will result in the improvement and standardization of multidisciplinary patient management and patient selection following current international best practices, leading to an increase in the LAPC resection rate in the Netherlands from 8 to 16% among patients who begin with multi-agent induction chemotherapy (m)FOLFIRINOX and/or gemcitabine with nab-paclitaxel. As a result, striving for the following primary and secondary targets:

Primary outcomes including target:

1. *Overall survival:* In patients after surgical resection, a median OS of 25 months, 1-year OS of > 90%, and 5-year OS of > 20%, measured from time of diagnosis. The OS will be compared to patients diagnosed with borderline resectable pancreatic cancer who underwent surgical resection after neoadjuvant (m)FOLFIRINOX in the PREOPANC-2 trial (EudraCT: 2017–002036-17) [46].
2. *In-hospital/30-day morbidity and mortality*: In patients after surgical resection, we aim for in-hospital/30-day mortality of ≤ 5% and in-hospital/30-day major morbidity of < 50%.

Secondary outcomes including target:

1. *Quality of life, mental and physical health status, and potential side effects on the short- and long-term*: In all patients included in the PREOPANC-4 cohort, a non-significantly different quality of life, mental and physical health status, and potential side effects, as compared to the following two cohorts:
  Patients diagnosed with borderline resectable pancreatic cancer who started with neoadjuvant (m)FOLFIRINOX in the PREOPANC-2 trial (EudraCT: 2017–002036-17) [46, 47].
  Patients from the historical cohort of patients with LAPC having RECIST non-progressive disease following 2 months of induction treatment with (m)FOLFIRINOX or gemcitabine with nab-paclitaxel chemotherapy (2015–2020) included in the PACAP cohort (NCT03513705) [48, 49].
2. *Healthcare satisfaction:* In all patients included in the PREOPANC-4 cohort, a non-significantly different patients’ healthcare satisfaction, as compared to the patients in the following two cohorts:
  Patients diagnosed with borderline resectable pancreatic cancer who started with neoadjuvant (m)FOLFIRINOX in the PREOPANC-2 trial (EudraCT: 2017–002036-17) [46, 47].
  Patients from the historical cohort of patients with LAPC having RECIST non-progressive disease following 2 months of induction treatment with (m)FOLFIRINOX or gemcitabine with nab-paclitaxel chemotherapy (2015–2020) included in the PACAP cohort (NCT03513705) [48, 49].
3. *Radical (R0) resection rate:* In patients after surgical resection, we are aiming for a non-significantly different radical resection (R0) rate as compared to the patients diagnosed with borderline resectable pancreatic cancer who underwent surgical resection after neoadjuvant (m)FOLFIRINOX in the PREOPANC-2 trial (EudraCT: 2017–002036-17) [46, 47].

The benchmarks for in-hospital/30-day major morbidity and mortality are established by the protocol group based on the international benchmarks for pancreatoduodenectomy with portomesenteric venous resection and the additional risks of major morbidity and mortality of arterial resections [50, 51]. The benchmarks for OS were established by the protocol group based on the international literature [15].

The resection rate among patients with LAPC (DPCG criteria) who started with induction chemotherapy (i.e., received at least 1 cycle of [m]FOLFIRINOX or gemcitabine with nab-paclitaxel) during the PREOPANC-4 implementation (2022–2024) will be compared with a recent Dutch cohort (2015–2017) of patients diagnosed with LAPC (DPCG criteria) who started with induction chemotherapy (i.e., received at least 1 cycle of [m]FOLFIRINOX or gemcitabine with nab-paclitaxel).

### Study population

All consecutive adult patients (i.e., ≥ 18 years) diagnosed with pathology-confirmed LAPC following the DPCG criteria are eligible to receive care according to the designed clinical work-up (see Fig. 1). Subsequently, the following patients meet the eligibility criteria for inclusion in the PREOPANC-4 cohort.

#### Inclusion criteria

1. Age ≥ 18 years;
2. Pathology confirmed LAPC according to the DPCG criteria (see Appendix 2 for the resectability criteria);
3. Non-progressive disease based on computed tomography (CT) following RECIST (version 1.1) [31] after a minimum of 4 months of chemotherapy with (m)FOLFIRINOX and/or gemcitabine with nab-paclitaxel.

### Study procedures

#### Informed consent

Informed consent will be obtained for using individual patient data for study purposes, in addition to the Dutch LAPC Registry [17, 33]. The format written informed consent will be obtained during or following (outpatient) clinic appointments by a member of the local study team (e.g., local researcher, research nurses, treating physician). The written informed consent will be obtained once the patient meets the eligibility criteria. Patients will be informed that study team members will review their medical records and that their data will remain confidential. Moreover, patients are asked for permission for the use of their data for scientific research. Participants will be notified that participating in this study does not affect the routine care that they will receive and that they are free to withdraw from the study at any time. All patients participating in PREOPANC-4 will be asked to participate in the Dutch LAPC Registry and the PACAP registry (NCT03513705) [48, 49]. However, it is expected that most patients will already join at that time as the aim is to enrol patients in both the LAPC Registry and PACAP project at the time of diagnosis (see section ‘*Data collection and management’*).

#### Follow-up

The follow-up strategy (e.g., cross-sectional imaging, tumour markers, and frequency) will not be standardized, as progression- or recurrence-free survival are no study endpoints.

#### Data collection and management

Data collection will be conducted through the prospective LAPC Registry [17, 33], capturing clinicopathological disease characteristics, details on surgical and oncological treatment, and surgical and survival outcome. In addition, surgeons will be asked to register intraoperative procedural details immediately after surgery via an electronic case report form using Castor Electronic Data Capture (Castor EDC). Prospective assessment of quality of life, functioning, adverse events/symptoms, and healthcare satisfaction is secured via the PACAP registry. Within the PACAP, patient-reported outcome measures (PROMs) are collected via (e)mail at the time of diagnosis, followed by assessments at 3, 6, 9, 12, 18, and 24 months, followed by life-long annual evaluations (NCT03513705) [48, 49]. Quality of life, functioning, and adverse events/symptom scores will be investigated using the EQ-5D-5L [52], EORTC QLQ-C30 (version 3.0) and QLQ-PAN26 [53, 54], Hospital Anxiety and Depression Scale (HADS) [55], and Worry of Progression Scale (WOPS) [56–58] questionnaires. Healthcare satisfaction will be measured using the Assessment of Patient Experiences of Cancer Care (APECC) and the EORTC QLQ-C30 questionnaires [53, 59]. The study will follow the FAIR principles (i.e., Findable, Accessible, Interoperable, and Reuseable) in handling and storage of data [60]. All data will be stored in an online secured database at the Amsterdam UMC. All patient data will be coded by an individual study number that will be used to connect the different datasets. Data Monitoring Committee is not needed as the Medical Research Involving Human Subjects Act (WMO) does not apply to the PREOPANC-4 program (W21_487 # 21.541).

### Definitions

Patient performance and comorbidity status will be defined following the Eastern Cooperative Oncology Group (ECOG) classification, American Society for Anesthesiologists (ASA), and the age-adjusted Charlson Comorbidity Index (CCI) [61–63]. Imaging-based resectability status will be classified according to the DPCG criteria (see Appendix 2) [20]. Clinical and pathological disease staging will follow the Tumour, Node, and Metastasis (TNM) classification by the American Joint Committee on Cancer (AJCC) [64].

The type of pancreatic resection and concomitant extended resections will be defined following the International Study Group for Pancreatic Surgery (ISGPS) [65]. In-hospital / 30-day major morbidity will be defined as Clavien-Dindo grade IIIa or higher [66]. Pancreatic surgery-specific complications, including postoperative pancreatic fistula, postpancreatectomy hemorrhage, delayed gastric emptying, chyle leak, and bile leak, will be classified following the ISPGS and International Study Group of Liver Surgery (ISGLS) guidelines, with grades B and C being considered as clinically relevant [67–71]. Organ failure will be defined as meeting at least one of the following criteria: at least 24 h of (1) respiratory insufficiency requiring intubation, (2) hemodynamic instability requiring inotropics, and/or (3) renal insufficiency requiring dialysis. The resection margin (i.e., R status) is defined according to the Royal College of Pathologists (i.e., R0: ≥ 1 mm margin; R1: < 1 mm margin clearance), except the anterior margin for which the 0 mm rule will be used [72].

The OS will be measured from the date of pathology-based diagnosis. In case of an imaging-based diagnosis of LAPC due to inconclusive pathology obtained via fine-needle aspiration or biopsy, the date of diagnosis is based on the date of first cross-sectional imaging, with the requirement for inclusion in PREOPANC-4 that the pathology-based diagnosis of pancreatic adenocarcinoma is verified later during surgery or after surgery. Disease recurrence after surgical resection is defined as the date of imaging-based suspicion of locoregional recurrence and/or distant metastases.

### Statistical considerations

Based on a recent cohort of patients with LAPC in the Netherlands and the expected increase in the resection rate due to the PREOPANC-4 implementation strategy [17], we expect that at least 223 patients will be included during the 3-year inclusion period, of whom at least 55 will undergo resection after chemotherapy.

For the assessment of the resection rate during the PREOPANC-4 implementation period compared to a historical Dutch cohort, a sub-group analysis will be performed with stratification for patients diagnosed with LAPC according to the National Comprehensive Cancer Network (NCCN) criteria (version 2.2024) [73].

The OS will be estimated using the Kaplan–Meier method, measured from the date of pathology-based diagnosis until the last follow-up or date of death by any cause. The OS will be presented for the overall study cohort and stratified for surgical exploration with the intention of resection. To compare the OS in patients who underwent resection within the PREOPANC-4 versus patients who underwent resection following neoadjuvant (m)FOLFIRNOX within the PREOPANC-2 trial, a Cox regression model will be used, adjusting for pre-specified confounders known prior to surgery, comprising (1) baseline characteristics at time of diagnosis including age, ECOG performance status, CCI, (2) disease characteristics at time of diagnosis including resectability status following the DPCG criteria, serum carbohydrate antigen 19–9 (CA19-9) preferably measured with non-elevated bilirubin, tumour location (i.e., pancreatic head versus body/tail), largest solid tumour size, clinical nodal stage (i.e., cN0 versus cN1-2) [64], any arterial involvement (i.e., superior mesenteric artery, celiac axis, and/or hepatic artery), (3) preoperative treatment characteristics including the chemotherapy regimen(s) and duration, (4) disease characteristics at restaging after neoadjuvant/induction chemotherapy including RECIST response and serum CA19-9 including the relative response [31]. A time-dependent Cox regression analysis, with left-truncation for the time between diagnosis and resection, is used to adjust for potential heterogeneity in the time intervals between diagnosis and surgery between the PREOPANC-2 trial and PREOPANC-4 program, which may cause immortal time bias. Adjustment for only preoperative confounders is performed so the findings can be used for preoperative counseling. To compare the R0/R1 rate in patients who underwent resection within the PREOPANC-4 versus patients who underwent resection following neoadjuvant (m)FOLFIRINOX within the PREOPANC-2 trial, a logistic regression model will be used to adjust for the same preoperative confounders. For this logistic regression model, eventual R2 resections will be included in the R1 group. For the Cox and logistic regression models, multiple imputation will be used for missing data at random.

The in-hospital/30-day major morbidity and mortality will be analysed for the overall number of patients who underwent resection, including sub-group analyses on patients with or without NCCN LAPC at diagnosis and types of surgical procedures including the type of pancreatectomy and extended resections. In addition, 90-day mortality from surgery will be presented.

The questionnaire-specific manuals will be followed to analyse the quality of life, functioning, symptoms/adverse events, and healthcare satisfaction. To investigate these outcomes over time and compare them with the PREOPANC-2 cohort, linear mixed models will be used, adjusting for the same covariates as in the Cox regression model. Multiple imputation will be used for missing data at random. Estimated marginal means and adjusted differences in these outcomes over time will be presented. In addition, the clinical significance of any potential differences between the PREOPANC-4 and the PREOPANC-2 trial will be analysed and presented from the EORTC QLQ-C30 and -PAN26 questionnaires [74]. A sub-group analysis will be performed for patients who underwent surgical resection after induction chemotherapy.

Data analyses will be performed under supervision of a statistician (M.A.). Statistical significance is considered as a two-tailed *P*-value of < 0.050.

### Patient-centered care

To optimally support the process of shared decision-making about medical and surgical treatment of LAPC, it is crucial to obtain more insight into the treatment preferences, values, and experiences of patients and their relatives and the treatment preferences of healthcare providers. With this information, decision support tools can be developed to improve the shared decision-making.

First, a qualitative interview study with patients (and their relatives) will be performed to identify patients’ values, preferences, and experiences about their disease (i.e., LAPC), eventual treatment journey and shared decision-making. Patients and their relatives will be interviewed (1) at baseline (i.e., at diagnosis or shortly after that), (2) after 4 months or after completing induction chemotherapy, and (3) 2 months thereafter or in case of surgery one month postoperatively. Patients diagnosed with LAPC and present in Amsterdam UMC, RACU, and Erasmus MC will be screened for participation, regardless of tumour-directed treatment (type). A sample of 20–25 patients (and their relatives) is aimed for, depending on the data saturation. The interviews will be analysed using thematic analysis [75].

Second, an interview study will assess the treatment preferences of healthcare providers (i.e., pancreatic surgeons, medical oncologists, and nurse specialists) from each participating DPCG centre, including at least one pancreatic surgeon, medical oncologist, and nurse specialist per centre. In the interviews, healthcare professionals will be presented with case vignettes for treatment decision-making at different moments during a treatment trajectory. Additional questions are used to investigate the participants’ minimally desired survival benefit of various treatment options. Quantitative data will be analysed using descriptive statistics.

Based on the results from both interview studies and with the use of service design methodology, we will develop textual and visual tools to support the process of patient counselling and shared decision-making [76, 77].

## Discussion

PREOPANC-4 is the first nationwide program designed to standardize and implement the international multidisciplinary best-practice for LAPC, addressing a need highlighted by previous nationwide studies [17, 18]. To support this effort, the DPCG invited 4 expert surgeons from internationally renowned LAPC centres of excellence to act as proctors.

Within the PREOPANC-4 program, the clinical work-up regarding disease staging, induction therapy, response evaluation, and selection criteria for surgery will be standardized. The design of this clinical work-up is based on evidence and on the practices and experiences of the proctors. The design of this clinical work-up does not preclude the possibility of equally effective or alternative approaches. For instance, biology-based response evaluation and subsequent selection for surgery is established in the PREOPANC-4 protocol on a relative serum CA19-9 response of more than 30% [78]. However, diverging evidence exists about the optimal relative and absolute serum CA19-9 cut-offs [79, 80]. Furthermore, another modality to assess biological disease response following induction chemotherapy is fluorodeoxyglucose positron emission tomography (FDG-PET) with CT or magnetic resonance imaging (MRI) [81–83]. As the evidence about the interpretation of FDG-PET findings for response evaluation following chemotherapy in patients with pancreatic cancer is still premature, FDG-PET is only considered routine practice in the PREOPANC-4 program for patients with non-elevated serum CA19-9 levels at diagnosis. On the other hand, other expert centres have standardized the use of FDG-PET for response evaluation following chemotherapy [84]. As part of the anatomy, biology, and condition-related selection criteria for surgery, condition is based on the surgeon’ estimation. More objective criteria could be incorporated in the future, considering the expanding evidence on body composition parameters in response to chemotherapy and the value of prehabilitation [85–87]. The PREOPANC-4 clinical work-up is considered as a dynamic implementation strategy that can be adjusted constantly, based on new evidence and experiences.

The value of radiotherapy, in addition to induction chemotherapy for patients with LAPC, is still under debate. Preoperative radiotherapy is discouraged in the PREOPANC-4 because there is no evidence of OS benefit [5]. Moreover, there is a possible risk for arterial hemorrhages and pseudoaneurysm after preoperative radiotherapy when an arterial resection or divestment is needed [88, 89].

The PREOPANC-4 program aims to facilitate the exchange of expertise on surgical techniques and intraoperative decision-making. Expanding the indications for surgery, including performing more advanced pancreatic resections, requires years of surgical training, as shown by international centres of excellence [36, 37, 90]. Therefore, the surgical indications will gradually expand which is expected to continue after the 3-year implementation phase. The training phase started in March 2021, followed by the implementation phase that started in January 2022.

In summary, the PREOPANC-4 program aims to implement the multidisciplinary best-practice for patients with LAPC on a nationwide scale and in a patient-centred way in collaboration with 4 international experts. Clinical targets have been identified.

### Trial status

In progress.

## Supplementary Information

Supplementary Material 1. Appendix 1. SPIRIT 2013 checklist. Appendix 2. DPCG criteria for locally advanced pancreatic cancer.

## Data Availability

The datasets generated and analysed during the PREOPANC-4 study are not publicly accessible, but are available on reasonable request via the corresponding author.

## Abbreviations

ASA: American Society for Anesthesiologists
CCI: Charlson Comorbidity Index
CT: computed tomography
DPCG: Dutch Pancreatic Cancer Group
ECOG: Eastern Cooperative Oncology Group
FDG-PET: fluorodeoxyglucose positron emission tomography
ISGLS: International Study Group for Liver Surgery
ISGPS: International Study Group for Pancreatic Surgery
LAPC: locally advanced pancreatic cancer
(m)FOLFIRINOX: a (modified) chemotherapy combination of 5-fluorouracil, leucovorin, irinotecan, and oxaliplatin
MRI: magnetic resonance imaging
NCCN: National Comprehensive Cancer Network
OS: overall survival
PROMS: patient-reported outcome measures
RECIST: response evaluation criteria in solid tumours
TNM: Tumour, Node, and Metastasis
